# Single-cell RNA-seq analysis and cell-cluster deconvolution of the human preovulatory follicular fluid cells provide insights into the pathophysiology of ovarian hyporesponse

**DOI:** 10.3389/fendo.2022.945347

**Published:** 2022-10-21

**Authors:** Kristine Roos, Ilmatar Rooda, Robyn-Stefany Keif, Maria Liivrand, Olli-Pekka Smolander, Andres Salumets, Agne Velthut-Meikas

**Affiliations:** ^1^ Department of Chemistry and Biotechnology, Tallinn University of Technology, Tallinn, Estonia; ^2^ Nova Vita Clinic AS, Tallinn, Estonia; ^3^ Division of Obstetrics and Gynecology, Department of Clinical Science, Intervention and Technology, Karolinska Institutet and Karolinska University Hospital, Stockholm, Sweden; ^4^ Department of Obstetrics and Gynaecology, Institute of Clinical Medicine, University of Tartu, Tartu, Estonia; ^5^ Competence Centre on Health Technologies, Tartu, Estonia

**Keywords:** ovarian sensitivity index, IVF, hyporesponse, preovulatory follicle, bulk RNA-seq, single-cell RNA-seq, granulosa cells, deconvolution

## Abstract

Reduction in responsiveness to gonadotropins or hyporesponsiveness may lead to the failure of *in vitro* fertilization (IVF), due to a low number of retrieved oocytes. The ovarian sensitivity index (OSI) is used to reflect the ovarian responsiveness to gonadotropin stimulation before IVF. Although introduced to clinical practice already years ago, its usefulness to predict clinical outcomes requires further research. Nevertheless, pathophysiological mechanisms of ovarian hyporesponse, along with advanced maternal age and in younger women, have not been fully elucidated. Follicles consist of multiple cell types responsible for a repertoire of biological processes including responding to pituitary gonadotropins necessary for follicle growth and oocyte maturation as well as ovulation. Encouraging evidence suggests that hyporesponse could be influenced by many contributing factors, therefore, investigating the variability of ovarian follicular cell types and their gene expression in hyporesponders is highly informative for increasing their prognosis for IVF live birth. Due to advancements in single-cell analysis technologies, the role of somatic cell populations in the development of infertility of ovarian etiology can be clarified. Here, somatic cells were collected from the fluid of preovulatory ovarian follicles of patients undergoing IVF, and RNA-seq was performed to study the associations between OSI and gene expression. We identified 12 molecular pathways differentially regulated between hypo- and normoresponder patient groups (FDR<0.05) from which extracellular matrix organization, post-translational protein phosphorylation, and regulation of Insulin-like Growth Factor (IGF) transport and uptake by IGF Binding Proteins were regulated age-independently. We then generated single-cell RNA-seq data from matching follicles revealing 14 distinct cell clusters. Using cell cluster-specific deconvolution from the bulk RNA-seq data of 18 IVF patients we integrated the datasets as a novel approach and discovered that the abundance of three cell clusters significantly varied between hypo- and normoresponder groups suggesting their role in contributing to the deviations from normal ovarian response to gonadotropin stimulation. Our work uncovers new information regarding the differences in the follicular gene expression between hypo- and normoresponders. In addition, the current study fills the gap in understanding the inter-patient variability of cell types in human preovulatory follicles, as revealed by single-cell analysis of follicular fluid cells.

## Introduction

The mechanisms of suboptimal ovarian response remain unsolved at the molecular level for many women up to 40 years of age who are undergoing assisted reproductive treatment (ART) with exogenous gonadotropins. These women experience low success rates with IVF due to a low number of retrieved oocytes (1, 2). Up to 30% of patients are affected by this clinical phenomenon of hyporesponse to ovarian stimulation (3). Moreover, excessive use of gonadotropins in stimulation may result in ovarian hyperstimulation syndrome, a potentially lethal condition characterized by the increased permeability of the vasculature and the development of ascites. In severe cases enhanced hemoconcentration leading to oliguria can be diagnosed (4). Therefore an optimal and safe stimulation regimen is crucial for all IVF patients (5). Various studies have aimed to assess the hyporesponsiveness by investigating patients’ ovarian sensitivity or resistance to gonadotropin stimulation (6, 7), screening the single nucleotide polymorphisms in the gonadotropin hormone receptors (8–10), and measuring serum concentrations of anti-FSH antibodies (11). The ovarian sensitivity index (OSI) has been launched as a parameter describing a patient’s reproductive potential to produce oocytes as a response to exogenous gonadotropin stimulation. OSI is a function that connects the total amount of exogenous gonadotropins used and the number of oocytes retrieved as a result of hormone stimulation (12). Although OSI correlates with many ovarian responsiveness biomarkers, like age, antral follicle count, and anti-Müllerian hormone (13, 14), to confirm OSI reliability for hyporesponders, the relationship with other factors must be thoroughly studied. Knowledge of the contributing factors affecting OSI has great importance for the improvement of the success rate of IVF treatment for the hyporesponders.

The gonadotropins follicle-stimulating hormone (FSH), and luteinizing hormone (LH) are essential for the expansion of the preovulatory follicle and ovulation. The diameter of a preovulatory follicle reaches up to 25 mm and it is surrounded by the basal membrane that separates theca cells from the internal structure with a fluid-filled cavity, consisting mostly of different types of granulosa cells (GCs) as well as a minority of other somatic cell types (15). The fluid-filled antrum divides the GCs into two major populations: mural and cumulus cells, each with distinct roles and RNA profiles (16, 17). Cumulus GCs are in direct contact with the oocyte and are responsible for the trafficking of metabolites between the two cell types (18) as well as for the meiotic resumption of the oocyte (19).

GCs in large preovulatory follicles express both FSH and LH/hCG receptors, whereas theca cells primarily express LH/hCG receptors (20, 21). Accordingly, both cell types play a vital role in regulating gonadotropin responses in the ovarian follicle. GCs proliferate actively to lead to the expansion of the follicle (22), liquid infiltration, vascularization (23), and the production and transport of hormones and metabolites into the preovulatory follicle to accomplish the meiotic maturation of the oocyte (24, 25). Furthermore, various publications have demonstrated morphological differences between individual GCs suggesting that GCs may contain multiple subpopulations with potentially distinct functional properties (26–28). Besides, it has been shown in model organisms that the mitotic activity and steroidogenesis of GCs are also affected by their location in the follicle and distance from the oocyte (29). Molecular communication shuffling by extracellular microRNA molecules between somatic cell types has been recently proposed to have importance to normal follicular function (30). Taken together, these cells play a key regulatory role in ovarian function, and a shift in their gene expression may affect their responsiveness to gonadotropins.

So far, studies have identified novel ovarian somatic cell clusters from preantral follicles (31), cumulus-oocyte complex (32), whole ovarian tissue (33), ovarian cortex (34), and also from the preovulatory follicular fluid (35), where the differentiation of somatic cells has culminated before ovulation. However, there is a knowledge-gap regarding the characterization of GCs, specifically from the preovulatory follicular fluid of patients classified as hyporesponders based on OSI. Here, we combine bulk RNA sequencing (RNA-seq) with single-cell RNA-seq (scRNA-seq) to explore changes in gene expression and the proportions of individual somatic cell clusters between well-characterized hypo- and normoresponders, by analyzing the cellular content of the follicular fluid. As a result, we highlight the molecular alterations that could potentially help to improve the outcome of hormone stimulation.

## Materials and methods

### Ethics statement

The study was approved by the Research Ethics Committee of the University of Tartu (approval no 289/M-8). Signed informed consent was obtained from all participants.

### Patients and sample collection

Female patients and oocyte donors undergoing IVF at the Nova Vita Clinic were enrolled in the study from September to December 2019. Patients were classified as hyporesponders (HR) based on hormone stimulation if they administered ≥200 IU of recombinant FSH (rFSH) to receive an oocyte. Of the recruited 80 patients, 46 were found to adequately respond to stimulation (normoresponders, NR), and 34 were HR. The average age of recruited patients was 32.9 ± 4.8 years (range 22-40) and the BMI was 22.3 ± 3.1 kg/m^2^ (range 17.0-34.5). All the recruited women had two ovaries. Nineteen women were eligible for RNA-seq analysis due to the availability of a sufficient number of follicular cells and the high quality of the extracted RNA (see below). The final cohort recruited for RNA-seq consisted of 10 NR and 9 HR patients. The NR group consisted of 4 oocyte donors and 6 patients with male factor infertility. The HR group included 1 oocyte donor and 8 patients with different infertility diagnoses. The causes of infertility among all eligible participants were distributed as follows: male factor only (n=6), tubal factor only (n=4), combination of tubal and male factor (n=1), multiple female factors (n=2), and unexplained (n=1), while five women were oocyte donors. Oocyte donors were excluded from the analyses regarding IVF and embryo transfer outcome, as all their oocytes were frozen. In the remaining NR group, 10 embryo transfers were performed resulting in 5 successful deliveries. In the HR group, the numbers were 10 and 4, respectively. All the 19 women satisfied the following criteria: age ≤ 40; BMI between 17-33; antral follicle count ≥ 5; and nonsmokers. Women with polycystic ovary morphology and other ovarian morphological abnormalities detected by ultrasound examination were excluded. Preovulatory follicle count was performed two days before the oocyte retrieval.

### Ovarian stimulation

All patients were treated with gonadotropin-releasing hormone (GnRH) antagonist (Cetrotide, Merck, Darmstadt, Germany) protocol and the ovarian stimulation was thereafter accomplished by administering rFSH (Gonal-f^®^, Merck or Puregon, N.V. Organon, Oss, The Netherlands) at a daily dose. Ovulation was triggered with 0.2 mg human chorionic gonadotropin (hCG) (Ovitrelle^®^, Merck, or Diphereline^®^, Ipsen Pharma Biotech, Paris, France) if at least two leading follicles reached 18 mm in diameter. Ovum pick-up (OPU) was scheduled 36 hours later and follicular fluid from preovulatory follicles with diameters >18 mm was aspirated. Only material visibly clear of blood contamination was used in the study.

### OSI calculation

OSI was calculated by dividing the total administered rFSH dose in IU by the total number of oocytes retrieved at OPU, thus obtaining the rFSH-to-oocyte ratio.

### Isolation and fixation of cells from the follicular fluid

Following the removal of the oocyte-cumulus complex, the follicular fluid was centrifuged for 10 minutes at 300g to isolate all cells. The cell pellets from multiple follicles were pooled to obtain enough follicular cells from every patient. Next, the cell pellets were washed with 1x DPBS + 0.04% BSA (DPBS/BSA) (DPBS, Corning Life Sciences, Tewksbury, California, USA; BSA, MilliporeSigma, Burlington, Massachusetts, USA), and the erythrocytes were lysed with Red Blood Cell lysis buffer (150 mM NH4Cl, MilliporeSigma; 10 mmol NaHCO3, and 1.3 mM EDTA, both Amresco Inc, Solon, Ohio, USA). The remaining cell mixture was centrifuged and resuspended in DPBS/BSA. Cells were treated with 200 µL hyaluronidase (FertiPro NV, Beernem, Belgium) and 5U of DNase I (Thermo Fisher Scientific, Waltham, Massachusetts, USA) for 30 minutes to break the extracellular matrix surrounding the cells, filtrated through a 40 μm filter (pluriSelect Life Science, Leipzig, Germany) to remove cell clumps, washed with DPBS/BSA, and fixed in 80% methanol (Naxo Ltd., Tartu, Estonia). Cell counting was performed with a hemacytometer (The Paul Marienfeld GmbH & Co, Lauda-Königshofen, Germany). Cells from each patient were divided into two parts: at least 5x10^4^ cells were used for bulk RNA-seq and >2.5x10^4^ cells for scRNA-seq. Cells were stored at -80°C until further processing.

### Bulk RNA extraction and quality control

Methanol-fixed cells were equilibrated to 4°C, centrifuged for 5 minutes at 750g, rehydrated in Wash-Resuspension Buffer (0.04% BSA MilliporeSigma; 1mM DL-Dithiothreitol Solution, Invitrogen, Waltham, Massachusetts, USA; 0.2 U/μl Protector RNase Inhibitor, Thermo Fisher Scientific; 3x SSC Buffer, Naxo Ltd.), and lysed in 700µL QIAzol solution (Qiagen, Germantown, Maryland, USA). Methanol fixation and rehydration were performed according to the protocol approved by 10X Genomics (36). Bulk RNA was extracted from pooled cells of individual patients, with miRNeasy Micro kit (Qiagen), according to the manufacturer’s instructions. The quality and concentration of purified RNA were evaluated on 2100 Bioanalyzer with the RNA 6000 Pico kit (Agilent Technologies, Santa Clara, California, USA). Samples with RNA integrity number (RIN) ≥ 7 were considered eligible for further analysis. In total, the cells from 9 NR and 9 HR patients were used for further bulk RNA-seq.

### Bulk RNA-seq and data analysis

Sequencing libraries from purified RNA were prepared with the QuantSeq 3’ mRNA-Seq Library Prep Kit FWD (Lexogen GmbH, Vienna, Austria). Samples were indexed to allow for multiplexing. Library quality and size range was assessed using 2100 Bioanalyzer with the DNA 1000 kit (both Agilent Technologies). The libraries were diluted to a final concentration of 2 nM and subsequently sequenced on an Illumina HiSeq4000 platform. Single-end reads of 50 bp length were produced with a minimum of 2M reads per sample. Quality control of raw reads was performed with FastQC v0.11.7 (37). Adapters were filtered with ea-utils fastq-mcf v1.05 (38). Using HiSAT2 (39), split-aware alignment was accomplished against the human reference genome hg19. Reads mapping to multiple loci in the reference genome were discarded. The resulting BAM files were handled with Samtools v1.5 (40). The reads per gene were quantified with HT-seq Count v2.7.14 (41). Count-based differential expression (DE) analysis was done with the R-based Bioconductor package DESeq2 version 1.34.0 (42). Reported p-values were adjusted for multiple testing with the Benjamini-Hochberg procedure (43), which controls the false discovery rate (FDR). Principal component analysis was used to inspect sample- and group-specific variation with the R package DESeq2 (42) using the top 500 most variable genes across all samples. Surrogate variable analysis with the Bioconductor package sva version 3.42.0 (44) was used to determine the age groups to perform relevant age adjustment in DE analysis. Raw sequencing data is available at the European Nucleotide Archive, accession no PRJEB50778.

### Single-cell RNA-seq and data analysis

The follicular cells from 3 NR patients were used for scRNA-seq analysis (**Supplementary Data 1**). Two of the patients overlapped with the bulk RNA-seq dataset described above. Methanol-fixed cells were equilibrated to 4°C, centrifuged for 5 minutes at 750g, rehydrated in Wash-Resuspension Buffer, passed through 40μm Flowmi Cell Strainer (SP Bel-Art, Wayne, New Jersey, USA), counted, and finally adjusted to a concentration of 1000 cells per microliter. The single-cell suspension was loaded onto the Chromium Controller (10x Genomics, Pleasanton, California, USA) and scRNA-seq libraries were generated by using the Chromium Controller Single-cell 3’ Kit v3.1 (10x Genomics) according to the manufacturer’s protocol. At least 6 000 cells were aimed to be analyzed per sample. The library quality and size range were assessed using a Bioanalyzer (Agilent Technologies) with the High Sensitivity kit. Illumina Library Quantification Kit (KAPA Biosystems Inc., Wilmington, Massachusetts, USA) was used for the final library quantification. Libraries were sequenced on an Illumina NovaSeq6000 platform according to the manufacturer’s recommendations. Pair-end reads of 28bp Read 1 for cell barcode and UMI, 8bp I7 index for sample index, and 91bp Read 2 for transcript were produced with a minimum of 250M reads per sample. BCL files produced by Illumina sequencers for each flowcell were demultiplexed based on the sample index and converted into FASTQ files using the Cell Ranger mkfastq function of Cell Ranger version 3.0.2 (10X Genomics). Using Cell Ranger count, the FASTQ files were aligned against the human reference genome (hg19) and annotated with the corresponding GTF file (release 93). Raw sequencing data is available at the European Nucleotide Archive, accession no PRJEB50778.

The filtering process and cell-cluster annotation were performed using Seurat version 4.0.5 (45). Cells were retained for further analysis in cases 1) the number of detected genes was 200-6000, 2) the proportion of mitochondrial genes was <10%, and 3) the proportion of hemoglobin genes was <5%. After filtering, 24 213 cells in total were subjected to further analysis. Batch effects between the patients were eliminated using the Harmony package (46). For normalization and scaling of the data, the scTransform (47) function was used, and cell clusters were identified using the FindClusters function (resolution 0.5, with 16 dimensions, original Louvain algorithm) and visualized using 2D uniform manifold approximation and projection (UMAP).

BAM files of the sequencing data are available at the European Nucleotide Archive, project accession no PRJEB50778, sample accession numbers ERR8521472, ERR8521473, and ERR8521474.

### Determining the differentially expressed genes in cell clusters and functional enrichment analysis

The FindAllMarkers function in Seurat was used to list the statistically significant differentially expressed genes (DEG) for each cell cluster and the FindMarkers function was used to compare the selected clusters. As a result, cell clusters were annotated according to the DEGs using information from The Human Protein Atlas database (48) as well as from the literature. Pathway enrichment analysis was performed with DEGs found in bulk RNA-seq and scRNA-seq analysis of obtained clusters. Queries of DEGs of interest were loaded into g:Profiler (Ensembl version 104, Ensembl Genomes version 51) (49) for the Reactome analysis to study the potential functions. Pathways for which the adjusted p-value [Benjamini-Hochberg FDR (43)] was <0.05 were considered statistically significantly enriched.

### Estimating cell fractions and imputing cell-cluster-specific gene expression

CIBERSORTx (50) was used to estimate the proportions of cell clusters identified by scRNA-seq from the bulk RNA-seq samples of individual patients. A signature single-cell expression matrix of each cell cluster was generated with S-batch correction that removed variances between different library preparation protocols. The permutation value for obtaining statistical results was set to 1000. The relative abundance of cell cluster fractions was calculated as an average of 7 runs (7000 total permutations) and cell cluster differences between the study groups were analyzed with a linear regression model adjusted for age.

CIBERSORTx group-mode was implied to impute a single representative gene expression profile of cluster 1 vs other clusters from a group of HR and NR bulk RNA-seq mixture samples. As a result, unfiltered cell-cluster-specific gene expression values were obtained for both study groups. Gene expression values of “0” were replaced with the existing minimum value and expression level differences between the HR and NR groups were analyzed on log_2_-transformed data.

### Statistical analysis

The patients’ clinical characteristics were described as mean ± standard deviation (SD). The continuous variables of OSI were log-normalized, and normal distribution was checked using the Shapiro-Wilk test (51). For comparisons between the characteristics of HR and NR patient groups, Student’s t-test was used. Metaphase II (MII) oocyte rate was calculated as the number of MII oocytes per retrieved oocytes. Fertilized oocyte rate was normalized for the number of MII oocytes. Good-quality embryos were defined as those where either 1) ≥6 blastomeres were present and embryo fragmentation was <50% on day 3; or 2) the size and the assessment of the inner cell mass and trophectoderm development were graded ≥1BB on day 5 or 6. The good-quality embryos meeting these criteria were either transferred and/or vitrified. The good-quality embryos were vitrified using VitriFreeze ES and thawed in VitriThaw ES (both FertiPro NV). The good-quality embryo rate was calculated according to the number of successfully fertilized oocytes. The cumulative live birth rate was calculated as the total number of deliveries (>28 weeks of gestation) divided by the total number of performed embryo transfers, including all fresh and the subsequent frozen-thawed cycles. The delivery of a singleton, twin, or other multiples was considered as one delivery. Linear regression was used to analyze the impact of age and OSI on different IVF cycle outcomes and the estimated cell fractions, except for cumulative live birth rate, where the Wilcoxon rank-sum test was used. Pearson correlation analysis was used to correlate clinical characteristics and estimated cell fractions. All statistical analyses were conducted using R software version 4.0.1 (52) in Windows 10 operating system. P-value <0.05 was considered statistically significant except for RNA-seq studies, where Benjamini-Hochberg FDR (43) <0.05 was used as a cut-off for reporting statistically significant results.

## Results

### Higher age is associated with reduced sensitivity to stimulation

**Figure 1:**
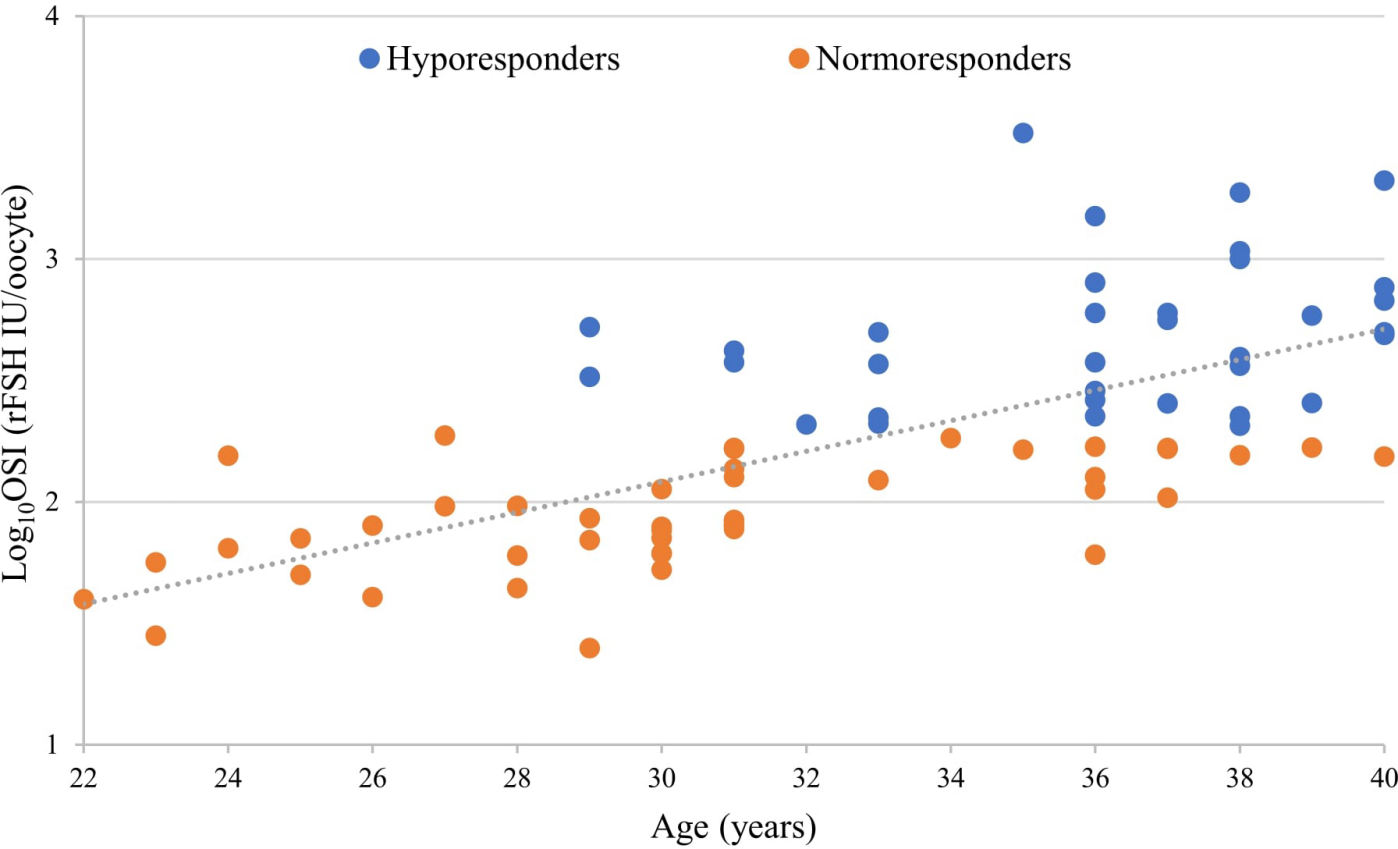
Correlation between the ovarian sensitivity index (OSI), a variable related to ovarian responsiveness, and patient’s age displayed a positive linear relationship (overall p<0.0001, overall R=0.673, Pearson correlation).

OSI links the number of retrieved oocytes to the units of administered rFSH, reflecting the intensity of the hormone stimulation response during the IVF treatment. Despite the dose adjustments, a significant proportion of patients exhibit lower responses. The condition is caused by multiple factors that require further clarification. Advanced age, as one of the factors, has been known to be directly related to poor stimulation outcomes (53). Therefore, we first assessed the characteristics of OSI and age in all 80 study participants stratified into HR and NR groups. The HR group was defined by a threshold value of OSI as ≥200 IU (log-transformed ≥2.301) of administered rFSH per oocyte. rFSH dosage of 150 IU per day during ovarian stimulation has been considered as the standard normal dosage by multiple studies (54, 55). Since there are no standardized criteria for the OSI formula and the threshold value for an impaired ovarian response, we decided to use the OSI ≥200 IU of rFSH per oocyte as a cut-off to define hyporesponsiveness in our study cohort. Such an approach connects higher-than-standard doses to oocyte yield. Unsurprisingly, in our study population, there was a positive correlation (**Figure 1**) determined between age and OSI (R=0.673, p=8.227*10^-12^). However, the correlation was statistically significant only in the NR (R=0.610, p=6.923*10^-06^) and not in the HR group (R=0.272, p=0.119). This leads to the hypothesis that age is not the only underlying factor in the ovarian response to rFSH hyperstimulation.

**Table 1** presents the characteristics and IVF outcome parameters of the recruited patients. The HR group was 5.5 years older on average (p<0.05) than the NR group. As expected, the HR group received increased dosages of administered rFSH, while the number of preovulatory follicles and retrieved oocytes were lower (all with p<0.05). The rFSH dose at the starting day of the ovarian stimulation was comparable between groups. The higher total rFSH amounts used for HR women derive from an increase in the dosage during days 5-10 and longer overall stimulation length (both p<0.05, **Supplementary Figure 1**) due to the weaker ovarian responsiveness of this patient group. The mean OSI in the HR group was 6.6 times higher (p<0.05) than in the NR group. Remarkably, the HR group had a higher rate of total good-quality embryos (p<0.05), but there was no statistically significant difference in the rate of metaphase II oocytes and fertilized oocytes between the study groups. The cumulative live birth rate was in a strong negative correlation with the OSI after adjustment with age (p=0.007, **Table 2**). On average the HR patients achieved live birth nearly twice less frequently than the NR women, but due to omitting oocyte donors from this comparison, this difference was not statistically significant (p=0.103, age-adjusted Wilcoxon rank-sum test, **Table 1**).

**Table 1:** Characteristics of the recruited study participants (n=80).

	Normoresponders (n = 46)				Hyporesponders (n = 34)				Age-adjusted p
	**Mean**	**SD**	**MIN**	**MAX**	**Mean**	**SD**	**MIN**	**MAX**	
**Age (years)**	30.6	4.5	22	40	36.1	3.2	29	40	**<0.001**
**BMI (kg/m²)**	21.6	2.4	17.0	27.7	23.2	3.8	18.7	34.5	0.190
**Administered rFSH (IU)**	1504.1	414.0	950.0	2925.0	2510.6	736.3	1125.0	4201.8	**<0.001**
**Preovulatory follicle count (n)**	19.8	9.6	7	50	7.6	3.8	1	14	**0.001**
**Retrieved oocytes (n)**	17.9	8.0	8	40	5.8	3.3	1	16	**<0.001**
**OSI (rFSH IU/oocyte)**	100.3	46.9	25.0	187.5	659.7	652.5	206.3	3300.0	**<0.001**
**Metaphase II oocyte rate (%)**	77.9	15.3	44.4	100.0	79.2	27.7	0.0	100.0	0.640
Fertilized oocyte rate (%)*	66.0	24.5	0.0	100.0	65.9	31.4	0.0	100.0	0.721
Good-quality embryo rate (%)*	40.2	24.2	0.0	85.7	57.5	33.9	0.0	100.0	**0.017**
Cumulative live birth rate (%)*	53.9	48.8	0.0	100.0	23.6	41.2	0.0	100.0	0.103

*Oocyte donors (n=18) are excluded from the calculation.

The metaphase II oocyte rate calculation was adjusted for the number of retrieved oocytes, fertilized oocyte rate for the number of metaphase II oocytes, and good-quality embryo rate for the number of fertilized oocytes. The cumulative live birth rate was calculated as the total number of deliveries (>28 weeks of gestation) divided by the total number of performed embryo transfers, including all fresh and the subsequent frozen-thawed cycles.

BMI, body mass index; rFSH, recombinant follicle-stimulating hormone; IU, international units; OSI, ovarian sensitivity index.

Values in bold imply statistically significant results between groups, age-adjusted p-value <0.05.

**Table 2:** Correlation between the ovarian sensitivity index (OSI) and other clinical factors (age-adjusted) of the recruited study participants (n=80).

	Coefficient	Adjusted R^2^	Age-adjusted p
**Age (years)**	7.195	0.446	**<0.001**
**BMI (kg/m²)**	1.759	0.068	0.088
**Preovulatory follicle count (n)**	-13.576	0.690	**<0.001**
**Metaphase II oocyte rate (%)**	0.085	-0.025	0.991
Fertilized oocyte rate (%)*	2.836	-0.025	0.781
Good-quality embryo rate (%)*	9.619	-0.019	0.391
Cumulative live birth rate (%)*	-45.888	0.234	**0.007**

*Oocyte donors (n=18) are excluded from the calculation.

The metaphase II oocyte rate calculation was adjusted for the number of retrieved oocytes, fertilized oocyte rate for the number of metaphase II oocytes, and good-quality embryo rate for the number of fertilized oocytes. The cumulative live birth rate was calculated as the total number of deliveries (>28 weeks of gestation) divided by the total number of performed embryo transfers, including all fresh and the subsequent frozen-thawed cycles. Values in bold imply statistically significant results between groups, age-adjusted p-value <0.05.

BMI, body mass index; rFSH, recombinant follicle-stimulating hormone; IU, international units; OSI, ovarian sensitivity index.

Age was positively correlated to OSI, whereas the preovulatory follicle count was observed to be negatively correlated to OSI with statistical significance (**Table 2**). All the above-mentioned correlations are strongly linked to indicators of ovarian potential.

### Hyporesponder and normoresponder patients exhibit different gene expression profiles in their preovulatory follicular fluid cells

Among the 18 patients selected for further RNA-seq experiments the above-described differences between the characteristics of the study groups remained valid (**Supplementary Data 2** **)**. RNA-seq of pooled cells isolated from follicular fluid (bulk RNA-seq) was performed for each patient to determine molecular mechanisms underlying the ovarian response to stimulation.

As demonstrated above, the underlying molecular processes behind hyporesponse to ovarian stimulation are affected and potentially masked by the effect of age. Furthermore, aging has a significant impact on the gene expression of follicular somatic cells (56, 57). In our bulk RNA-seq data, we found that the hidden source of variation in genome-wide gene expression correlates with age from 34 years and above (**Supplementary Figure 2**). As a result, age was treated as a binary parameter (≤33 years and older) in the relevant subsequent analyses.

**Figure 2:**
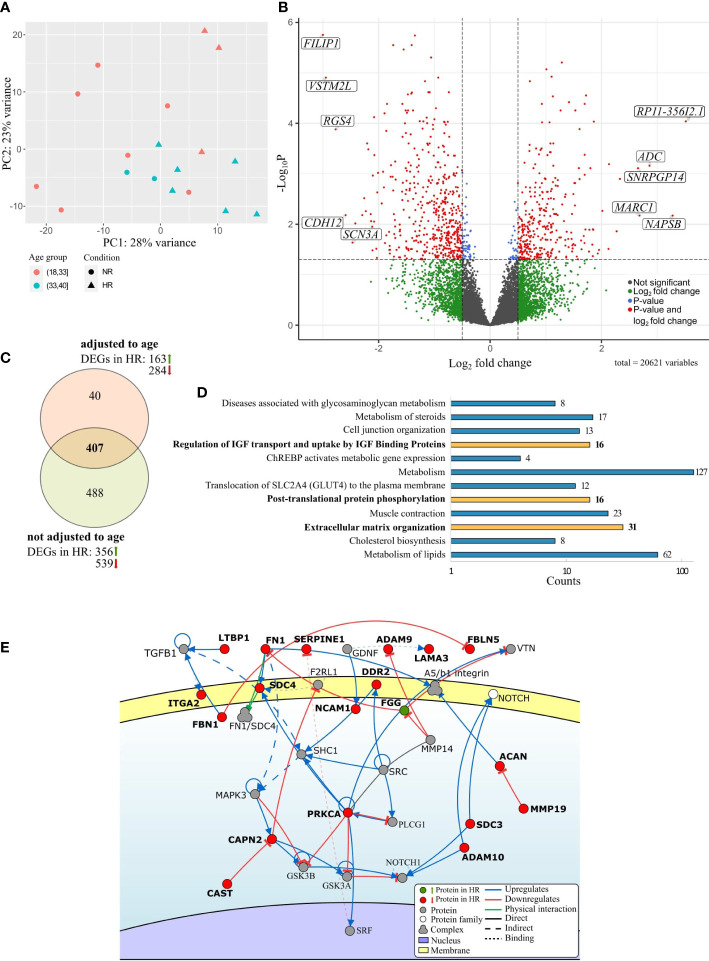
Overview of differentially expressed genes (DEGs) between the preovulatory follicular fluid somatic cells of hypo-(HR) and normoresponders (NR) identified by bulk RNA-seq. **(A)** Principal component analysis of the gene expression data from the follicular cells of HR (triangles) and NR patients (circles). Age groups (18-33 and 34-40 years) are marked in color. **(B)** Volcano plot highlighting top 5 upregulated and downregulated statistically significant DEGs between the HR and NR group, based on the fold change. **(C)** Venn diagram of DEGs between study groups with and without age adjustment. **(D)** Enrichment analysis of Reactome pathways based on DEG data between HR and NR groups (FDR<0.05) and presented in the order of decreasing statistical significance. Molecular pathways that remained significantly enriched after age adjustment are depicted by bold text and yellow bars. **(E)** A section of the major extracellular matrix organization pathway is shown schematically [adapted from Signor (58)]. Genes depicted in bold were differentially expressed between the follicular fluid somatic cells of HR and NR patients. The direction of gene expression difference between study groups is denoted by color: green indicating upregulation and red indicating downregulation in the HR group. Genes illustrated in grey were not differentially expressed.

Indeed, the principal component analysis demonstrated a clear separation between the bulk RNA expression profiles of HR and NR groups that was not explained by age difference alone (**Figure 2A**). To effectively comprehend the degree of age effect on gene expression variations between HR and NR groups, differential gene expression analysis of bulk RNA-seq data was conducted by using two statistical models: 1) without any adjustments and 2) with age adjustments in previously described groups.

Accordingly, if no age adjustments were done, 895 genes were found to have significantly different expression levels between the HR and NR groups (**Supplementary Table 1**; **Figure 2B**). After adjusting to age, 447 DEGs with statistical significance remained (**Supplementary Data 2**), with 407 genes shared by both statistical models (**Supplementary Tables 1** and **2** indicated in bold; **Figure 2C**). We conclude that these genes are involved in molecular processes that underlie hyporesponsiveness to gonadotropins age-independently.

The genes that were differentially expressed between the ovarian somatic cells of HR and NR groups were enriched into 12 Reactome pathways including lipid and steroid metabolism, as well as cholesterol biosynthesis, and cell junction organization (FDR<0.05). Importantly, three pathways remained significantly enriched regardless of age: extracellular matrix (ECM) organization, post-translational protein phosphorylation, and regulation of IGF transport and uptake by IGF Binding Proteins, the latter two sharing the DEG list **(** **Figure 2D**, **Supplementary Table 3**). The majority of the DEGs that regulate ECM organization such as *LAMA3*, *ITGA2*, *ACAN*, *ADAM9*, *ADAM10*, *FN1*, *PRKCA*, *FBN1*, and *SERPINE1* were downregulated in the HR group **(** **Figure 2E** **)**. The upregulation of FGG in HR patients may be one of the explanations for the reduction of functions for several other pathway members as *FN1* and its downstream targets **(** **Figure 2E** **)**. In conclusion, significantly altered gene expression affects the organization of the ECM and pathways related to IGF signaling in women with diminished response to gonadotropins.

### Somatic cell clusters in the preovulatory follicle

The gene expression dissimilarities in the follicular somatic cells revealed between HR and NR patients may be explained by the variation in the proportions of infiltrated immune cells or the unsimilar rate of differentiation/luteinization of GCs. Hence, we aimed to generate a single-cell transcriptome map of the preovulatory follicular cells from the follicular fluid based on fertile women from the NR group that can be further used for cell cluster deconvolution from bulk RNA-seq datasets of larger patient groups.

**Figure 3:**
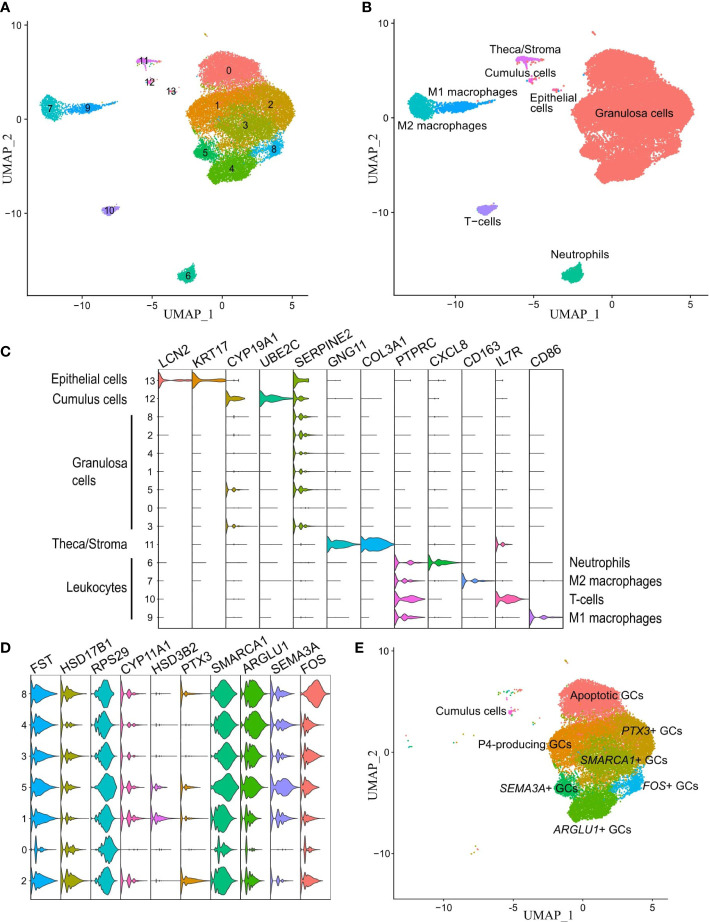
Unraveling cell clusters in the human preovulatory follicle. **(A)** Individual cell clusters detected from the single-cell RNA-seq dataset and analyzed with the original Louvain algorithm were visualized on UMAP. **(B)** The expression of known marker genes for each cell cluster group. **(C)** Annotated UMAP of the identified 4 leukocyte cell clusters, epithelial cells, theca/stroma cells, cumulus cells, and granulosa cells (GCs). **(D)** The presence of GC clusters was confirmed by the expression of known GC-specific markers (*FST*, *HSD17B1*). **(E)** Annotated clusters of GCs in the preovulatory follicle.

Single follicular somatic cells from 3 NR patients, including 2 women with male-factor infertility and 1 oocyte donor, were sequenced. The mean age of the patients was 31.0 ± 4.6, OSI 65.7 ± 27.4, and the cumulative live birth rate was 100% (**Supplementary Data 1** **)**. No segregation was observed according to the cell cycle phase or the source of cells from individual patients in the merged dataset (**Supplementary Figure 3**). In total, 24 213 single cells passed the quality filtering (**Supplementary Data 3**) and subsequently separated into 14 clusters (**Figure 3A**). To trace the distinct cell clusters and characterize their gene expression patterns, cluster-specific DEGs were obtained (Supplementary Data 4). Of note, some differences in the cell proportions across detected clusters were observed between individual patients (**Supplementary Table 4**).

We first identified 4 leukocyte lineages (PTPRC+, alternatively known as CD45+) that accounted for 11.3% of total cells, including neutrophils, T-cells, M1, and M2 macrophages. Next, 10 non-immune cell lineages (PTPRC-) were identified, including epithelial cells and theca/stroma cells, which together accounted for 1.2% of total cells, cumulus cells, and 7 clusters of GCs that accounted for 87.5% of total cells. Cell clusters were annotated based on known expressed markers summarized from literature and the Human Protein Atlas database (48): *CXCL8*, *MX2*, and *CSF3R* for neutrophils; *IL7R* for T cells, *CD86* for M1 macrophages; *CD14* and *CD163* for M2 macrophages (59); *CD46*, *KRT17* and *LCN2* for epithelial cells; *COL3A1*, *IGFBP5*, *GNG11*, *COL1A1* and *COL1A2* for theca/stroma cells (33, 60, 61); and *VCAN*, *CYP19A1*, and *UBE2C* for cumulus cells (33, 62) (**Figures 3B, C**). The remaining 7 unidentified clusters (numbers 0-5 and 8) formed a major group of cells marked by high expression of *SERPINE2*, *HSD17B1*, *CD59*, *FST*, *CDH2*, *PLA2G16*, and *AKIRIN1* (33), and a lack of markers for epithelial and cumulus cells. These clusters were collectively termed as GCs (**Figure 3C**) and studied further in more detail.

### Gene expression dynamics of granulosa cell clusters

The identified GC subtypes were characterized using two methods (1): highly expressed DEGs were identified for each GC cluster separately (**Supplementary Table 4** **)**; and (2) each GC cluster was compared to the pooled dataset of the remaining GC clusters (**Supplementary Table 5** **)**. Each GC cluster exhibited a unique set of highly expressed genes. Reactome enrichment analysis resulted in 368, 23, 16, 8, 331, 60, and 190 terms for GC clusters 0, 1, 2, 3, 4, 5 and 8, respectively **(** **Supplementary Table 6** **)**. Though the GC clusters varied in size, they were detected in all analyzed patient samples (**Supplementary Figure 4**).

It is crucial to be able to characterize the features of GC clusters to understand their impact on ovarian responsiveness to gonadotropin stimulation. We therefore carefully examined the gene expression profiles of all these clusters **(** **Figure 3D** **)**. Cluster 0 was distinguished from other GC clusters due to the high expression of apoptotic markers (such as *RPS29*, *UBB*, *UBC*, *UBA52*, and *RPS27A*) and a vast number of ribosomal genes. The DEGs are involved in the control of apoptosis, ubiquitination, and p53 signaling according to the Reactome pathway analysis.

GC cluster 1 was recognized by *FDX1* and *HSP90AB1* expression as well as high levels of *STAR*, *HSD3B2*, and *CYP11A1* – genes for the key enzymes in progesterone production. STAR initiates the transfer of cholesterol from high-density lipoproteins into mitochondria, where it is converted to pregnenolone by CYP11A1 and progesterone by HSD3B2. DEGs of cluster 1 participate in the metabolism of steroid hormones, cholesterol biosynthesis, and estrogen-dependent nuclear events downstream of ESR-membrane signaling. In GC cluster 2, *PTX3*, *MT2A*, *CTSC*, *CYB5A*, *INHA*, and *HSD17B1* were among the most highly expressed genes and showed features in the metabolism, post-translational protein phosphorylation, regulation of IGF transport, and uptake by IGF Binding Proteins, and VLDL assembly.

DEGs from GC cluster 3 such as *SMARCA1*, *PRKAR2B*, *PTGES*, *VCAN*, *INSR*, and *CALM1* are associated with Interleukin (IL)4 and IL13 signaling. Highly expressed *NEAT1*, *MALAT1*, *ARGLU1*, *TSHZ2*, *ADAMTS9*, and *LAMA3* in cluster 4 were linked to active participation in insulin receptor recycling and laminin interactions, metabolism of steroid hormones, and gluconeogenesis. Cluster 5 was distinguished by *SEMA3A*, *TECRL*, *INHBA*, and *ADAMTS1* expression as well as *ITGA2* which were related to the cell-extracellular matrix interactions and synthesis of very-long-chain fatty acyl-CoAs. Altogether, the findings of clusters 4 and 5 DEGs confirm that they have a relevant role in ECM remodeling and response to gonadotropin surges. GC cluster 8 displayed high expression of *FOS*, *JUN*, *DNAJB1*, *EGR1*, and *PTGS2* which are involved in signaling *via* NTRK1 (TRKA) and ILs.

Taken together, we were able to identify and characterize 7 GC clusters and named these accordingly: Apoptotic GCs (cluster 0), Progesterone-producing luteinized GCs (cluster 1, P4-producing GCs), *PTX3*+ GCs (cluster 2), *SMARCA1*+ GCs (cluster 3), *ARGLU1*+ GCs (cluster 4), *SEMA3A*+ GC (cluster 5), and *FOS*+ GC (cluster 8) (**Figures 3D, E**).

### Preovulatory follicles of hyporesponder patients contain fewer *ARGLU1*+ GCs, *SEMA3A*+ GCs, and theca/stroma cells

**Figure 4:**
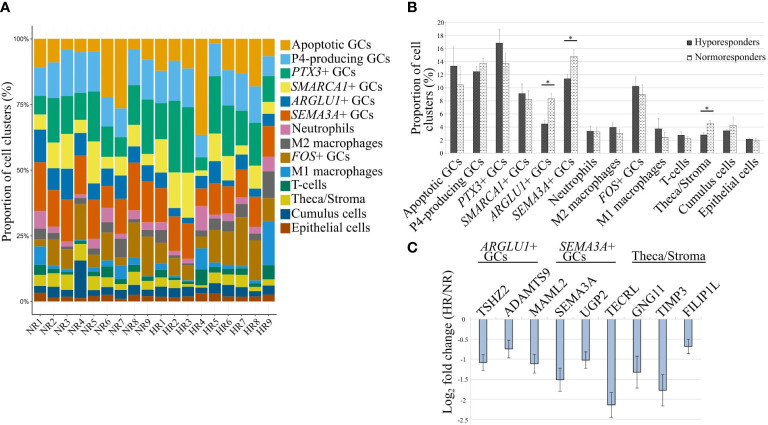
Changes in cellular proportions across identified clusters between hypo- and normoresponder patients (HR and NR, respectively). **(A)** The proportions of 14 cell clusters in 18 individual patients. **(B)** Cellular deconvolution of cell cluster proportions compared between the study groups. *- p-value <0.05 (age-adjusted linear regression). **(C)** The fold change of expression levels of 3 marker genes corresponding to the cell clusters with a statistically significant difference in their proportions between the HR and NR groups that are depicted in **(B)**.

With the single-cell analysis, we identified follicular cell clusters according to their gene expression profiles in the human preovulatory follicular fluid. Prognosis or manifestation of hyporesponse to gonadotropin stimulation in patients undergoing IVF treatment may be caused by either the variation in the proportion of these cell clusters or the gene expression differences in individual cell clusters without a change in cell proportions. To better understand the heterogeneity of individual cell clusters between patients and study groups, the CIBERSORTx computational framework was used on patients with available bulk RNA-seq data (n=18) to deconvolute the proportions of 14 cell clusters that were previously identified by scRNA-seq (**Figure 4A** **)**. Importantly, fewer *ARGLU1*+ GCs (p=0.018), *SEMA3A*+ GCs (p=0.005), and theca/stroma cells (p=0.021) were identified in the samples of HR patients in comparison to the NR group when adjusted to age (**Figure 4B**). In addition, we observed that the marker genes of these three GC clusters (**Supplementary Table 4**) were downregulated in the bulk RNA-seq data of the HR patients (**Figure 4C**; **Supplementary Table 1**). This observation confirms that a change in a cell cluster proportion is partly underlying the gene expression differences verified by bulk RNA-seq.

### Individual cell clusters contribute to the gene expression disturbances in hyporesponder patients

We further aimed to understand if the change in gene expression levels between HR and NR patients observed in bulk RNA-seq data is a contribution of individual cell clusters without a change in the cluster proportion. We observed that 9 marker genes of the P4-producing luteinizing GCs were differentially expressed between HR and NR patients and coincided with 8 Reactome pathways that were disturbed in correlation with the significant change in OSI values (**Figure 2D**; **Supplementary Tables 3** and **4**).

**Figure 5:**
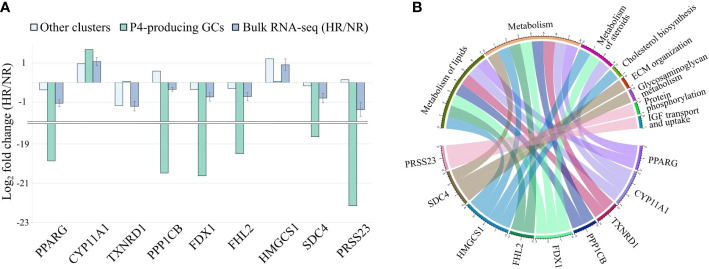
Influence of the P4-producing GC cluster (progesterone producing luteinized granulosa cells) on gene expression changes between hypo- (HR) and normoresponder (NR) groups. **(A)** Comparison of nine P4-producing GC-specific gene values in bulk RNA-seq and deconvoluted datasets. **(B)** Distribution of the nine P4-producing GC-specific genes between Reactome pathways affected by increasing OSI values.

To evaluate if the expression difference of these 9 genes between HR and NR patients is indeed specific for the P4-producing luteinized GCs, we recreated the gene expression profile of the P4-producing cluster in comparison to all other clusters from our study groups using the CIBERSORTx group-mode analysis. In conclusion, we observed that the expression of 8 genes out of 9 in the P4-producing luteinized GCs were consistent with those reported in bulk RNA-seq data (**Figure 5A**). Only the expression of *TXNRD1* in the P4-producing luteinized GC cluster was not consistent with the direction of expression differences observed in the bulk RNA-seq dataset between HR and NR patients. This observation suggests that the differential expression of *TXNRD1* in bulk RNA-seq results from expression in other cell clusters. Concordance of the other 8 genes implies that their overall downregulation in HR patients is affected by their fundamental shift of gene expression levels in the P4-producing luteinized GCs. Each of the nine evaluated genes is linked to a Reactome pathway *via* a chord diagram to characterize the affected biological processes **(** **Figure 5B** **)**.

In summary, the integration of bulk RNA-seq data with scRNA-seq through a deconvolution-based model has provided a novel understanding of the underlying mechanisms of hyporesponse. Our results suggest that variation in follicular cell cluster composition and altered gene expression in P4-producing luteinized GC clusters correlate with OSI values.

## Discussion

The consideration of high OSI as a predictive marker of ovarian hyporesponsiveness in IVF still needs to be clarified. The OSI parameter may be particularly helpful in counselling patients during their IVF treatment because high OSI expresses gonadotropin dosage amount, which can lead to complications such as ovarian hyperstimulation syndrome. Moreover, the OSI parameter defines the response to hormone stimulation by the number of oocytes retrieved that is linked to many IVF cycle outcomes such as embryo quality and thus live birth rate (63, 64). Unfortunately, the OSI value of the patient’s current IVF cycle becomes available only after the procedure. Therefore, expanding the described OSI concept by investigating contributing factors other than those previously mentioned, such as age, antral follicle count (13), and anti-Müllerian hormone (12) benefits to classify patients with lower ovarian response to hormone stimulation before they start treatment, enabling to adapt the doses of the stimulation drugs to be used.

Our work is the first to profile the direct association between the OSI parameter and genome-wide RNA expression level variations of the preovulatory follicular fluid cells of patients undergoing IVF.

Furthermore, we integrated scRNA-seq and bulk RNA-seq datasets of the isolated cells to dissect the differences in the gene expression and proportions of somatic cell clusters between HR and NR patients.

The OSI parameter consists of two variables: exogenous gonadotropin dose and the number of oocytes retrieved. Different formulas for OSI calculations and thresholds for determining hyporesponse have been used without a clear consensus. We calculated OSI as the total exogenous gonadotropin dosage used divided by the number of retrieved oocytes. The use of OSI ≥200 IU of rFSH per oocyte as a cut-off to define hyporesponsiveness was based on the knowledge that rFSH 150 IU per day is considered a standard treatment dose (54, 55) and hence, administrating ≥200 IU of rFSH per oocyte describes also a higher dose treatment for oocyte retrieval during IVF cycle. For instance, Huber et al. (65), used the OSI formula in which the number of recovered oocytes was multiplied by 1000 and divided by the total amount of administered rFSH. Patients were divided into three response groups based on the OSI levels: poor, normal, and high. Their cut-off level for a poor response was much higher in comparison to our study: it was defined as OSI <1.697/IU, which corresponded to 11 patients with OSI ≥600 in our study. Our analysis demonstrates that the somatic cells in the preovulatory follicle are affected already by milder gonadotropin stimulation excess than proposed by Huber et al.

We found no differences in the rates of metaphase II and fertilized oocytes between study groups, but the HR group had a higher rate of good-quality embryos and a lower cumulative live birth rate. Some studies show a decrease in oocyte quality, increased aneuploidy, and therefore a lower live birth rate in women with a poor response (66, 67), while others conclude the opposite (68, 69). One of the likely reasons for the contentious results is the accompanying influence of ovarian aging, which is difficult to eliminate due to the small number of women recruited for studies. Secondly, the inclusion criteria are different between studies: while some consider the stricter Bologna (70) or Poseidon (71) criteria for poor response, the hyporesponse is determined according to the efficiency of gonadotropin stimulation only. Regardless of this, it has been demonstrated that using low doses of rFSH during IVF improves the cycle outcomes, such as the rate of fertilization, embryo quality, and live birth, as well as the patient’s comfort during the therapy (72).

It is well established that a higher age contributes to lower ovarian response, increasing the rFSH dosage (73, 74) and resulting in a decline in reproductive potential (75, 76). Likewise in our study, age positively correlated with OSI; moreover, the correlation was statistically significant only in the NR group. Therefore, our study hypothesizes that if age is not currently the main indicator for HR, other contributing factors must also be assessed.

First, the study confirms that the gene expression profiles of the preovulatory follicular fluid cells between HR and NR are significantly different. We revealed three biological pathways that are significantly affected, regardless of age. These are ECM organization, post-translational protein phosphorylation, and regulation of IGF transport and uptake by IGF Binding Proteins. In the HR group, most of the genes in these detected pathways are downregulated. The latter finding is especially interesting as IGF-1 has been proposed as a potential target for individualized controlled ovarian stimulation strategy. Increasing IGF expression by using growth hormone as a supplement during ovarian stimulation may be useful for activating folliculogenesis in poor responder patients (77), as IGF-1 has synergistic effects with gonadotropins (78). However, more randomized clinical trials are still needed to prove the concept.

ECM is responsible for ovarian morphology as well as the signal transduction within the preovulatory follicle. The LH surge stimulates ovulation in GCs by activating numerous ECM re-organizing processes (79), including the signal transduction mechanism *via* binding of Sp1/Sp3 transcription factors (80). This process contributes to successful ovulation by occurring concurrently with active post-translational phosphorylation and glycosylation (81). The reduced expression of several members of the ADAM and ADAMTS metalloprotease families, such as *ADAMTS9*, *ADAM9*, and *ADAM10*, indicates a significant shortage of essential ovulatory mediators and lowered oocyte quality in HR patients (82, 83). Even more so, downregulated *ADAMTS-1*, a key gene in ECM organization, is an important mediator of LH and progesterone effects during ovulation (84), and its functional form is selectively localized on cumulus complex cell surfaces (85). Cumulus cells encircle oocytes and provide metabolites *via* gap junctions, influencing oocyte maturation and developmental competence (86). Likewise, post-translational protein phosphorylation is required in cumulus-oocyte complexes to mediate nuclear and cytoplasmatic maturation (87), while the disturbances of this process may contribute to aneuploidy or abnormal oocyte (88). These findings suggest that the development of a mature oocyte during folliculogenesis is highly dependent on these identified pathways. The application of bulk RNA-seq on the preovulatory follicular fluid cells from HR and NR groups delineated certain molecular alterations associated with HR. However, the bulk RNA-seq results cannot exclusively indicate whether differences shown in gene expression levels are the main reason for hyporesponsiveness. Alternatively, changes in preovulatory follicle cell cluster proportions may also contribute to this condition. Accordingly, we have described the single-cell transcriptomes of NR preovulatory follicles. Using the scRNA-seq dataset and the following deconvolution analysis, allowed us to investigate the cellular composition of HR preovulatory follicles and potentially reveal the underlying factors for the impaired response.

One of the main findings of our study is the identification of 14 cell clusters from the preovulatory follicles by their unique cell cluster-specific marker genes. In follicular fluid samples, we were able to identify four types of CD45+ leukocytes, theca/stroma cells, epithelial cells, cumulus cells, and 7 subtypes of GCs. The presence of epithelial cells (89) has been described previously, as also multiple types of immune cells have been found in ovarian follicular fluid (90–92). While the number of macrophages, T-lymphocytes, and NK cells in the follicular fluid have been associated with several aetiologies of infertility, no significant variability in the proportions of leukocyte clusters were observed in our study.

The role of GCs deserves to be explored in detail because disturbances in their function could be the cause of different female reproductive disorders. By determining the transcriptomes of the GC clusters, we were able to propose their molecular functions. The GCs are vigorously producing essential steroid hormones such as progesterone and estrogen, and we were able to characterize different intensities of steroidogenic capability in the GC clusters. Elevated activity of steroid synthesis was observed in clusters 1, 2, and 5. Notably, cluster 1 expressed all of the key enzymes required for progesterone production at detectable levels: STAR for transporting cholesterol from the outer to inner mitochondrial membrane; and CYP11A1 and HSD3B2 for converting cholesterol into progesterone (93), which is enhanced by the electron donor FDX1 (94). From the bulk RNA-seq data, we highlighted Reactome pathways, like the metabolism of lipids and steroids, as well as cholesterol biosynthesis altered in HR. Adding the scRNA-seq dataset layer allowed us to demonstrate for the first time that differences in the expression of the genes enriched into these pathways are specific for distinct GC clusters.

However, it needs to be emphasized that the scRNA-seq method does not reveal the transcriptome level at a comparable depth as bulk RNA-seq methods. Hence, our results do not claim that steroidogenic pathways are not present in other identified GC clusters, rather they were not observed at the current detection limit.

Focusing on transcriptomic patterns that distinguish GC clusters, we identified an apoptotic GCs cluster (cluster 0). An increased proportion of apoptotic GCs has a negative impact on the developmental potential of the oocyte and the subsequent embryos (95), by limiting the supply of metabolites and interfering with cell communication. Some studies have attempted to evaluate the apoptosis rate of the mural and cumulus GCs collected during OPU using several apoptosis markers staining and flow cytometry analysis to estimate the chance of IVF failure (96, 97). A high expression of *PTX3* and *CD24* was found in cluster 2 cells, suggesting that these cells participate in the reorganization of the hyaluronan matrix to initiate ovulation. Furthermore, the anti-inflammatory and angiogenesis-promoting features of *PTX3* indicate that these GCs act as a protective layer in the preovulatory follicle by regulating the inflammatory milieu and maintaining a balanced microenvironment (98, 99). Cluster 4 expresses *ARGLU1*, which is required for estrogen receptor-mediated gene transcription (100), as well as *LAMA3*, a laminin family member that participates in ovarian follicle ECM and cell junction organization, increasing GCs proliferation and survival as demonstrated in sheep (101). They provide enzymatic activity to the GCs in response to gonadotropin surges (102). *SEMA3A*, which has been shown in mice to play an important role in mediating luteinization processes following an LH surge (103), and *INHBA*, which has been shown in sheep to promote GCs proliferation, hormone synthesis, and inhibit apoptosis (104), are both highly expressed in cluster 5. By expressing a high level of *FOS* and *JUN*, cluster 8 shows features that participate in periovulatory processes with metabolic activities such as prostaglandin synthesis and cholesterol biosynthesis (105). These above-mentioned genes are involved in the downstream regulation of progesterone production and transport across GCs as well as EGF-signalling (106).

Interestingly, Wu et al. (35) have recently shown that they identified nine different functional clusters of GCs from follicular fluid cells. As there were no prior datasets available for GC clusters, we are both among the first to confirm that GCs divide into multiple clusters with different functions. Some GC clusters that appeared in both studies have similarly expressed genes. Additionally, we were able to identify cumulus cell cluster as well. Variations between studies may have arisen due to heterogeneity between the patients because of the small number of samples (3 vs 6) utilized. In addition, some technical differences were present between the two studies involving some details in sample processing and scRNA-seq library preparation protocols.

Understanding the difference in the proportions of cell clusters between HR and NR helps in the identification of cellular targets to improve IVF therapy and its outcomes. In this study, a computational approach combining data from scRNA-seq and bulk-RNA-seq, as well as cell deconvolution method CIBERSORTx (50) was used to reveal the different proportions of cell clusters between patient groups. We discovered that the proportions of three clusters: *ARGLU1*+ and *SEMA3A*+ GC clusters, along with theca/stroma cells are significantly under-represented in HR, coinciding with the lower gene expression values of the corresponding marker genes in the case of ovarian hyporesponse.

While the importance of *ARGLU1*+ and *SEMA3A*+ GC clusters in the development of hyporesponse needs further studies, there is already previous indication on the role of theca cells on the response to controlled ovarian stimulation (107). Although there is a lack of comprehensive assessment of theca cell proportions related to the ovarian stimulation response, it has been proposed that patients with theca cell shortage have decreased follicle structural support, and LH-stimulated androgen production (108). Serum androgen level is correlated with AFC, anti-Müllerian hormone, and thus to ovarian response to rFSH (109). Furthermore, it has been demonstrated that theca cells have a reduced capacity to respond to hCG/LH and the production of androgens decreases from the age of 30 (110). Authors of the latter study propose that this result derives from the change in the proportions of theca and granulosa cells during aging. Our findings reveal that patients with high OSI, regardless of age, have a lower number of theca cells.

In this study, we were able to demonstrate variations in the overall distribution of cell clusters and cell cluster-specific gene expression levels between the HR and NR groups. We confirmed that the deconvoluted data from bulk RNA-seq included our described marker genes retrieved by scRNA-seq verifying the reliability of such bioinformatic approach. Similar cell cluster-specific deconvolution analyses from bulk RNA-seq data have also been used in other biological systems: in onco-immunology the immune and cancer cell fractions have a major impact on the survival prognosis (111), and on the response to immunotherapy (112), or in unraveling novel cell types from whole tissue samples (113). The combination of affordable bulk RNA-seq data with the reference scRNA-seq datasets by cell cluster deconvolution method offers a cost-efficient approach for performing transcriptomic investigation on a large number of samples (114, 115). The type of analyses used in the current study allows for the generation of extensive novel information and clinically relevant associations from the datasets previously published in data repositories.

There are some limitations to our study. All our results were obtained from the bioinformatic analysis and experimental validation regarding the functionality of the identified cell clusters should be performed in the future. The presented datasets were generated by RNA-seq and the validation of the cell cluster markers at the protein level was not performed. The small study group size is another drawback. Further research on the additional clinical application is still needed. Nonetheless, patients, their data, and performed analyses serve as a foundation for investigating the differences between HR and NR at a single-cell level.

Collectively, the evidence proposed in this paper demonstrates that suboptimal results to ovarian stimulation could be associated with an altered cell-cluster composition or cell-cluster-specific gene expression changes in the preovulatory follicle. We have revealed molecular pathways which serve as potential prognostic biomarkers in the clinical management of ovarian hyporesponse. Investigating the underlying cause of the insensitivity of follicles to stimulation would allow identifying the suitable therapeutic targets to treat HR: either by modifying dysfunctional molecular pathways or by promoting cell cluster-specific differentiation potential. The findings of this study identify novel reasons for ovarian stimulation failure and propose directions for future research.

## Data availability statement

The raw data underlying the study is publicly available at the European Nucleotide Archive with the project accession number PRJEB50778.

## Ethics statement

The studies involving human participants were reviewed and approved by the Research Ethics Committee of the University of Tartu. The patients/participants provided their written informed consent to participate in this study.

## Author contributions

AV-M was responsible for the study’s conception and conduct. AV-M, AS and O-PS were responsible for study funding. KR was responsible for the sample collection. KR, IR, R-SK, ML, and AV-M analyzed and interpreted the data. KR and AV-M wrote the manuscript. All authors reviewed and approved the final version of the manuscript.

## Funding

EASI-Genomics - This project has received funding from the European Union’s Horizon 2020 research and innovation program under grant agreement No 824110 (EASI-Genomics PID:7712). In addition, the research was funded by the Estonian Research Council (grants PRG1076 and PSG608), Horizon 2020 innovation grant (ERIN, grant no. EU952516), and the Enterprise Estonia (grant no. EU48695). Olli-Pekka Smolander was supported by the “TTÜ development program 2016–2022,” project code 2014–2020.4.01.16–0032.

## Acknowledgments

We are extremely grateful to the personnel of Nova Vita Clinic, Tallinn, Estonia for their valuable contributions in discussing hyporesponse etiology and collecting samples, as well as to the patients who participated in the study. We would also like to thank Kristine Stepanyan and David Carbonez from Genomics Core Leuven for sharing their expertise in single-cell library preparation, sequencing, and primary quality control.

## Conflict of interest

The authors declare that the research was conducted in the absence of any commercial or financial relationships that could be construed as a potential conflict of interest.

## Publisher’s note

All claims expressed in this article are solely those of the authors and do not necessarily represent those of their affiliated organizations, or those of the publisher, the editors and the reviewers. Any product that may be evaluated in this article, or claim that may be made by its manufacturer, is not guaranteed or endorsed by the publisher.
