# Supplementary material for: Single-cell RNA-seq analysis and cell-cluster deconvolution of the human preovulatory follicular fluid cells provide insights into the pathophysiology of ovarian hyporesponse
Source: Front Endocrinol (Lausanne). 2022 Oct 21;13:945347. doi: 10.3389/fendo.2022.945347 (PMC9635625; doi:10.3389/fendo.2022.945347)
Supplement: Supplementary file 1 [file DataSheet_1.docx]

***Description of Supplementary Files***

File Name: **Supplementary Data Sheet 2**

Description: Contains supplementary data (tables) and figures in the same order as they are referred to in the manuscript.

File Name: **Supplementary Table 1**

Description: List of differentially expressed genes (FDR<0.05) identified by DESeq2 between the hypo- and normoresponder patient groups.

File Name: **Supplementary Table 2**

Description: List of differentially expressed genes (FDR<0.05) identified by DESeq2 between the hypo- and normoresponder patient groups adjusted to age.

File Name: **Supplementary Table 3**

Description: Reactome enrichment analysis of differentially expressed genes from without age-adjustment and age-adjusted analysis performed between hypo- and normoresponder patient groups. Shared genes are marked in bold.

File Name: **Supplementary Table 4**

Description: List of the most variably expressed genes per cluster (FDR<0.05, average log_2_ fold change > 0.25, positive value).

File Name: **Supplementary Table 5**

Description: List of marker genes per granulosa cell cluster (FDR<0.05) by comparing each granulosa cluster to the pool of remaining granulosa cells. Each comparison is presented on a separate sheet.

File Name: **Supplementary Table 6**

Description: Reactome enrichment analysis of differentially expressed genes between each granulosa cell clusters compared to the pool of remaining granulosa cells (the FindMarkers function). Reactome analysis result for each granulosa cell cluster is presented on a separate sheet.
