# Supplementary material for: Single-cell RNA-seq analysis and cell-cluster deconvolution of the human preovulatory follicular fluid cells provide insights into the pathophysiology of ovarian hyporesponse
Source: Front Endocrinol (Lausanne). 2022 Oct 21;13:945347. doi: 10.3389/fendo.2022.945347 (PMC9635625; doi:10.3389/fendo.2022.945347)
Supplement: Supplementary file 2 [file DataSheet_2.docx]

***Supplementary figures and data***

| **Supplementary Data 1. Characteristics of 3 normoresponder patients analyzed by scRNA-seq** | | | | |
| --- | --- | --- | --- | --- |
|  | **Normoresponders (n=3)** | | | |
|  | **Mean** | **SD** | **MIN** | **MAX** |
| **Age (years)** | 31.0 | 5.6 | 26 | 37 |
| **BMI (kg/m²)** | 21.5 | 2.9 | 19.8 | 24.8 |
| **Administered rFSH (IU)** | 1283.3 | 256.6 | 1000.0 | 1500.0 |
| **Preovulatory follicle count (n)** | 29.0 | 15.5 | 14 | 45 |
| **Retrieved oocytes (n)** | 23.0 | 12.5 | 13 | 37 |
| **OSI (rFSH IU/oocyte)** | 65.7 | 33.6 | 40.5 | 103.9 |
| **Metaphase II oocyte rate (%)** | 92.0 | 7.9 | 84.2 | 100.0 |
| **Fertilized oocyte rate (%)*** | 60.3 | 23.5 | 43.8 | 76.9 |
| **Good-quality embryo rate (%)*** | 72.9 | 18.2 | 60.0 | 85.7 |
| **Cumulative live birth rate (%)*** | 100.0 | 0.0 | - | 100.0 |
| *Oocyte donor (n=1) is excluded from the calculation.  The metaphase II oocyte rate calculation was adjusted for the number of retrieved oocytes, fertilized oocyte rate for the number of metaphase II oocytes, and good-quality embryo rate for the number of fertilized oocytes. The cumulative live birth rate was calculated as the total number of deliveries (>28 weeks of gestation) divided by the total number of performed embryo transfers, including all fresh and the subsequent frozen-thawed cycles. | | | | |
| BMI - body mass index, rFSH - recombinant follicle-stimulating hormone, IU - international units, OSI - ovarian sensitivity index | | | | |

| **Supplementary Data 2. Characteristics of the patients analyzed with bulk RNA-seq (n=18)** | | | | | | | | | |
| --- | --- | --- | --- | --- | --- | --- | --- | --- | --- |
|  | **Normoresponders (n=9)** | | | | **Hyporesponders (n=9)** | | | | **p-value** |
|  | **Mean** | **SD** | **MIN** | **MAX** | **Mean** | **SD** | **MIN** | **MAX** |  |
| **Age (years)** | 30.0 | 5.6 | 24 | 40 | 35.9 | 3.6 | 29 | 40 | **0.017** |
| **BMI (kg/m²)** | 21.3 | 3.3 | 17.0 | 26.6 | 23.4 | 4.1 | 19.8 | 32.7 | 0.244 |
| **Administered rFSH (IU)** | 1677.8 | 600.7 | 1150.0 | 2925.0 | 2726.2 | 802.5 | 1575.0 | 4201.8 | **0.006** |
| **Preovulatory follicle count (n)** | 20.2 | 9.9 | 14 | 45 | 9.3 | 3.3 | 4 | 14 | **0.007** |
| **Retrieved oocytes (n)** | 17.4 | 8.4 | 9 | 37 | 7.3 | 4.2 | 2 | 16 | **0.005** |
| **OSI (rFSH IU/oocyte)** | 109.7 | 45.4 | 40.5 | 166.7 | 555.6 | 594.9 | 206.3 | 2100.9 | **<0.001** |
| **Metaphase II oocyte rate (%)** | 87.5 | 8.0 | 73.7 | 100.0 | 87.7 | 14.2 | 57.1 | 100.0 | 0.969 |
| **Fertilized oocyte rate (%)*** | 73.4 | 16.5 | 60.0 | 100.0 | 56.9 | 33.9 | 0.0 | 100.0 | 0.336 |
| **Good-quality embryo rate (%)*** | 51.1 | 22.0 | 28.6 | 83.3 | 54.9 | 35.6 | 0.0 | 100.0 | 0.835 |
| **Cumulative live birth rate (%)*** | 60.0 | 41.8 | 0.0 | 100.0 | 47.6 | 50.4 | 0.0 | 100.0 | 0.804 |
| *Oocyte donors (n=5) are excluded from the calculation. | | | | | | | | | |
| The metaphase II oocyte rate calculation was adjusted for the number of retrieved oocytes, fertilized oocyte rate for the number of metaphase II oocytes, and good-quality embryo rate for the number of fertilized oocytes. The cumulative live birth rate was calculated as the total number of deliveries (>28 weeks of gestation) divided by the total number of performed embryo transfers, including all fresh and the subsequent frozen-thawed cycles. Values in **bold** imply statistically significant results between groups, p-value <0.05. | | | | | | | | | |
| BMI - body mass index, rFSH - recombinant follicle-stimulating hormone, IU - international units, OSI - ovarian sensitivity index | | | | | | | | | |


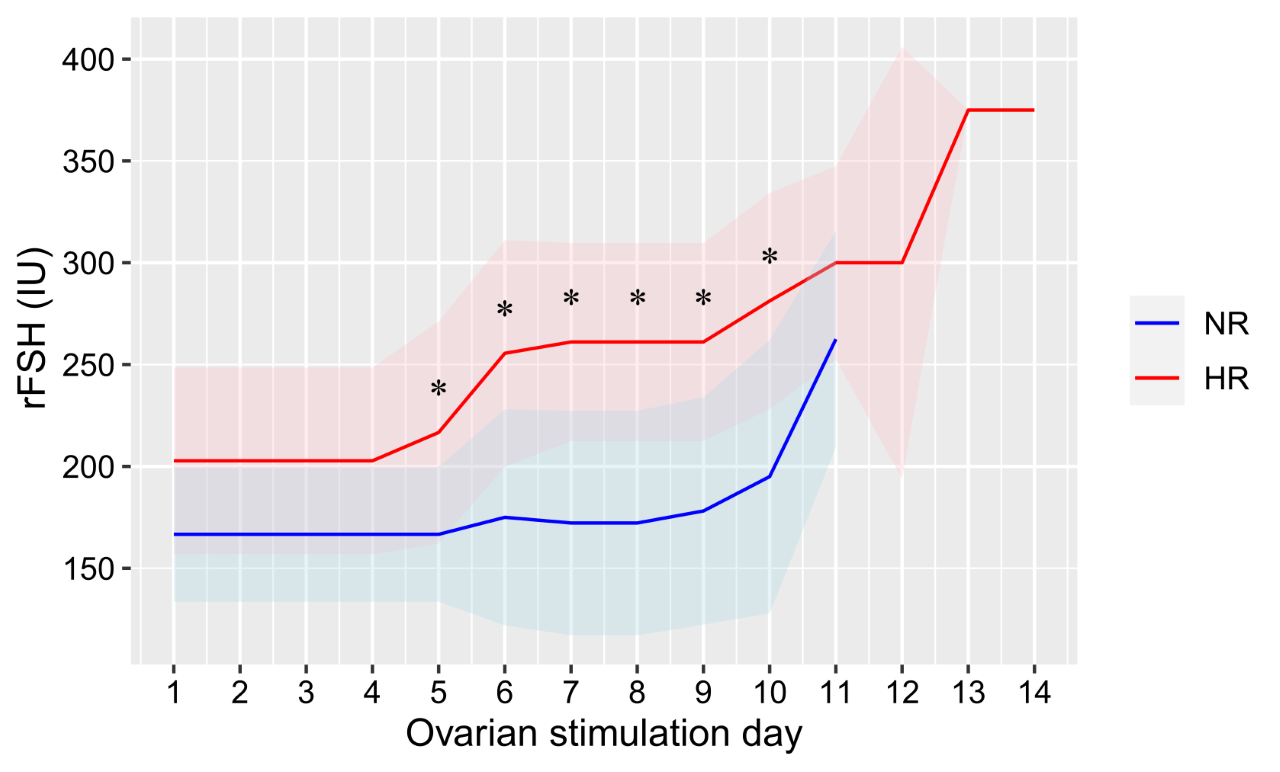


**Supplementary Figure 1.** Average daily doses of recombinant follicle-stimulating hormone (rFSH) used for hypo-(HR) and normoresponder (NR) patients during ovarian stimulation (N=18). The follicular cells of the same patients were used for RNA sequencing. Shaded areas correspond to 95% confidence intervals. * - p-value<0.05 between groups.

**
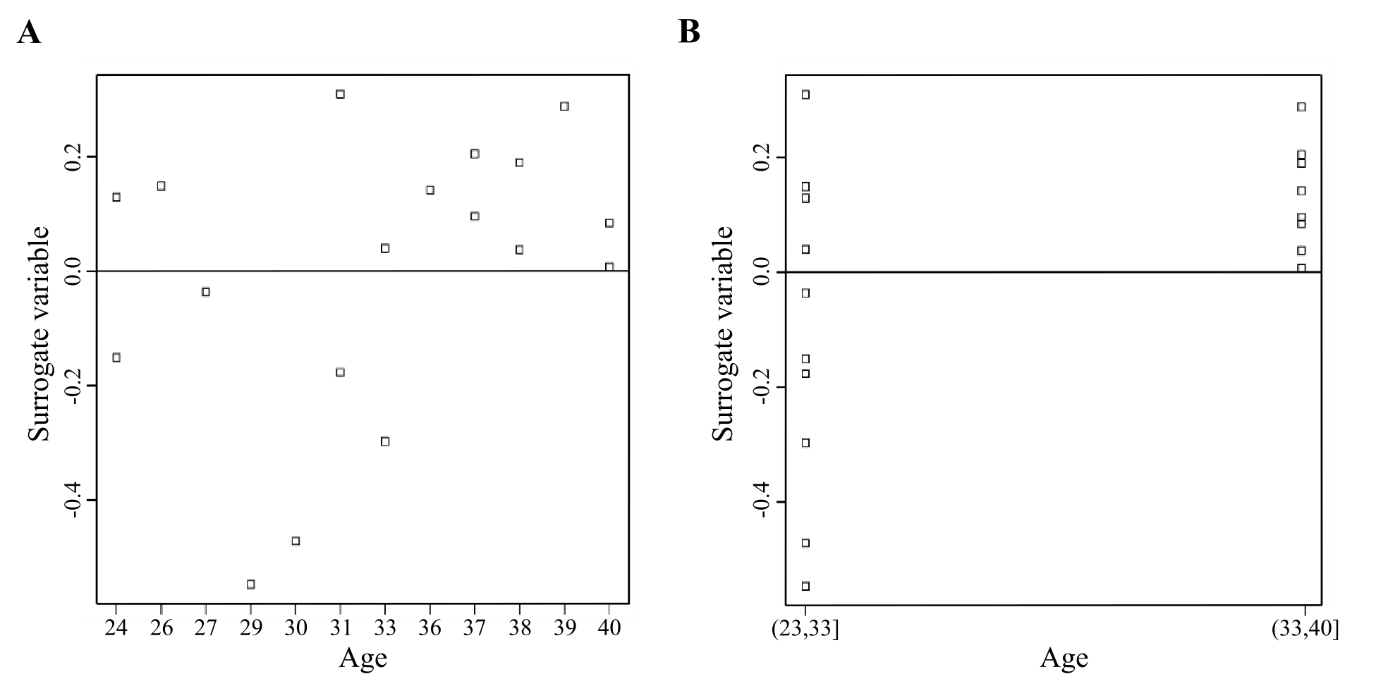
**

**Supplementary Figure 2.** The surrogate variable analysis (sva) demonstrates that age ≥34 years correlates with the hidden source of variation in gene expression data. **(A)** According to the analysis results **(B)**, splitting age groups between 33 and 34 years was performed.


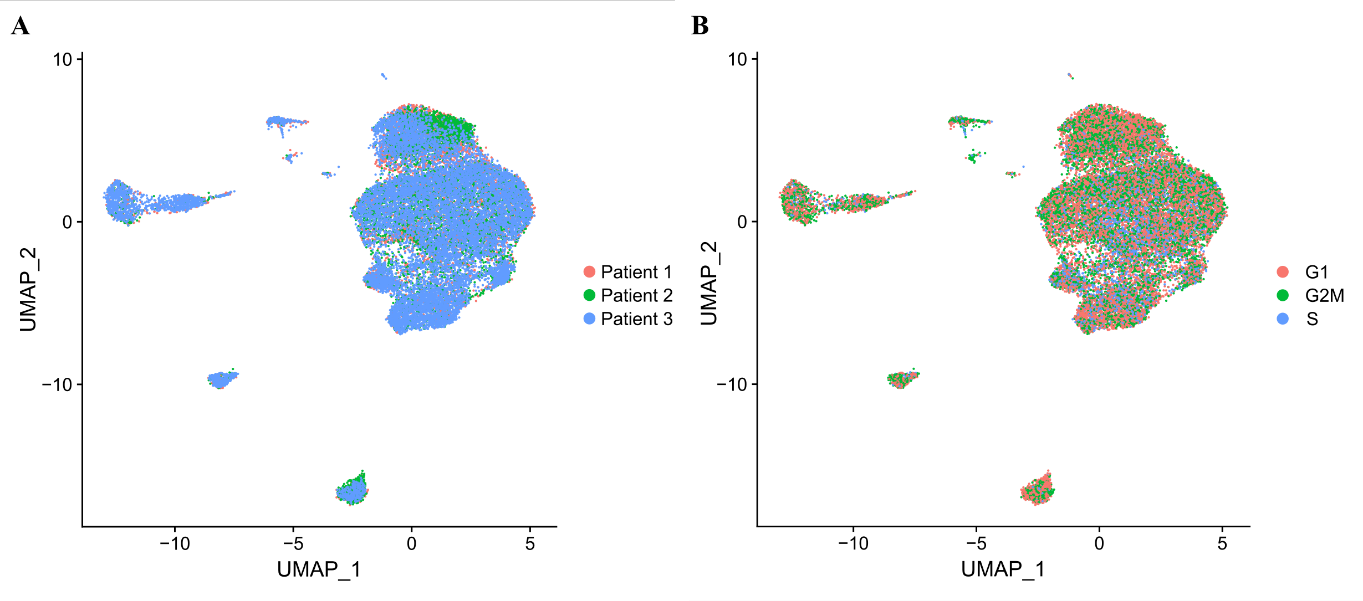


**Supplementary Figure 3**. The UMAP plot projections of individual cells by patient and cell cycle phases. **(A)** No segregation of individual patients into distinct cell clusters was observed. **(B)** Cell clusters displayed equal distribution between cell cycle phases.

| Supplementary Data 3. Single-cell sequencing quality and filtering parameters | | | |
| --- | --- | --- | --- |
| Sequencing results | **Patient 1** | **Patient 2** | **Patient 3** |
| Estimated Number of Cells | 6132 | 6062 | 13 763 |
| Mean Reads per cell | 52 154 | 36 517 | 22 171 |
| Median Genes per cell | 836 | 1466 | 1299 |
| Total Genes Detected | 20 562 | 20 527 | 22 086 |
| Sequencing Saturation | 66.2% | 49.8% | 52.3% |
| Filtering parameters: | (1) The total number of genes  detected in each cell, nfeature 200-6000  (2) The proportion of transcripts that are of mitochondrial origin < 10%  (3) The proportion of hemoglobin transcripts < 5% | | |
| Number of cells after quality filtering | 5817 | 5600 | 12796 |


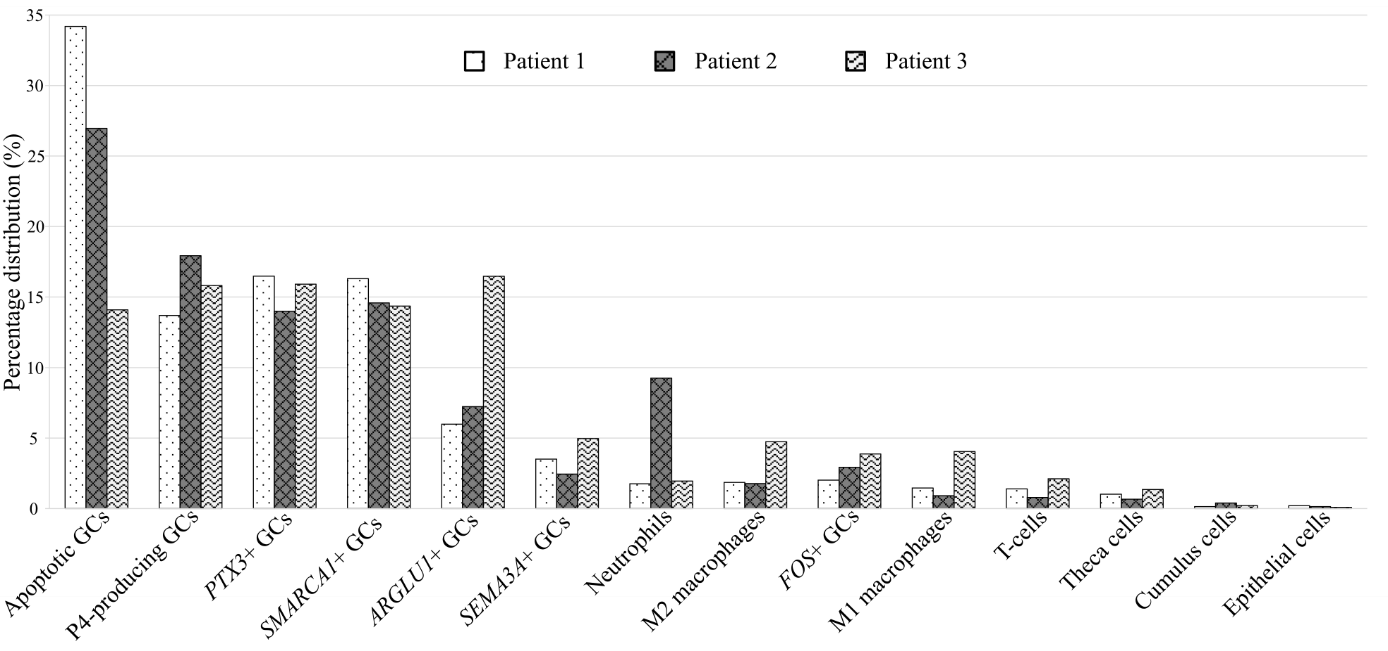


**Supplementary Figure 4.** The distribution of cell clusters between individual patients determined by single-cell RNA-seq. GCs – granulosa cells, P4 – progesterone.
